# Synergic Role of Dietary Bioactive Compounds in Breast Cancer Chemoprevention and Combination Therapies

**DOI:** 10.3390/nu16121883

**Published:** 2024-06-14

**Authors:** Marisabel Mecca, Marzia Sichetti, Martina Giuseffi, Eugenia Giglio, Claudia Sabato, Francesca Sanseverino, Graziella Marino

**Affiliations:** 1 Laboratory of Preclinical and Translational Research, Centro di Riferimento Oncologico della Basilicata (IRCCS-CROB), 85028 Rionero in Vulture, Italy; martina.giuseffi@crob.it (M.G.); eugenia.giglio@crob.it (E.G.); claudia.sabato@crob.it (C.S.); 2 Unit of Gynecologic Oncology, Centro di Riferimento Oncologico della Basilicata (IRCCS-CROB), 85028 Rionero in Vulture, Italy; francesca.sanseverino@crob.it; 3 Unit of Breast Cancer, Centro di Riferimento Oncologico della Basilicata (IRCCS-CROB), 85028 Rionero in Vulture, Italy; graziella.marino@crob.it

**Keywords:** phytochemicals, diet, breast cancer, cancer prevention, PUFA, bromelain, isothiocyanate, glucosinolates

## Abstract

Breast cancer is the most common tumor in women. Chemotherapy is the gold standard for cancer treatment; however, severe side effects and tumor resistance are the major obstacles to chemotherapy success. Numerous dietary components and phytochemicals have been found to inhibit the molecular and signaling pathways associated with different stages of breast cancer development. In particular, this review is focused on the antitumor effects of PUFAs, dietary enzymes, and glucosinolates against breast cancer. The major databases were consulted to search in vitro and preclinical studies; only those with solid scientific evidence and reporting protective effects on breast cancer treatment were included. A consistent number of studies highlighted that dietary components and phytochemicals can have remarkable therapeutic effects as single agents or in combination with other anticancer agents, administered at different concentrations and via different routes of administration. These provide a natural strategy for chemoprevention, reduce the risk of breast cancer recurrence, impair cell proliferation and viability, and induce apoptosis. Some of these bioactive compounds of dietary origin, however, show poor solubility and low bioavailability; hence, encapsulation in nanoformulations are promising tools able to increase clinical efficiency.

## 1. Introduction

Breast cancer (BC) is the second most common cause of cancer death in women [1,2]. Clinical and demographic indicators of BC prognosis include large tumor size, lymph node involvement, hormone receptor-negative subtype, diagnosis at an early age, and low socio-economic status [3]. Both genetic and environmental factors play a pivotal role in the risk of BC development among women [4].

BC is well known as a heterogeneous, complex disease with a spectrum of histopathological patterns and molecular characteristics due to genetic, epigenetic, and transcriptomic changes [5,6]. All this has led to a wide range of clinical findings, treatment responses, and clinical outcomes. Traditional classification criteria are based on biological features such as tumor size, lymph nodes involved, histological grade, and patient age and have limited the development of tailored treatment strategies so far [6]. Tumors with similar clinical and pathological conditions can often show different behaviors. To date, the molecular classification of tumors through detailed analysis of genetic changes and biological events involved in the initiation and progression of cancer has been extensively studied. In particular, BC heterogeneity is associated with differences or losses in molecular biomarkers: the amplification or overexpression of progesterone receptor (PR), estrogen receptor (ER), and human epidermal growth factor receptor 2 oncogene (HER2) [6,7,8,9,10]. About two-thirds of BC cases are ER^+^ and/or PR^+^, which are hormonally sensitive and responsive to endocrine therapy. Overexpression of the tyrosine kinase receptor HER2 has been frequently reported in hormone receptor-negative (HR^−^) compared to HR^+^ cancers correlated with hyperproliferation, aggressive behavior, and serious illness. Additionally, triple-negative breast cancer (TNBC), which accounts for almost 15% to 20% of BC cases, is clinically negative for ER, PR, and HER2 expression. TNBC has a damaging clinical behavior, frequently metastasizes, is difficult to treat, and does not respond to hormonal therapy or HER2 receptor-targeted therapy. Surgical resection, radiation, endocrine/hormonal therapy, chemotherapy, targeted therapy, or a combination of these approaches is used to treat and manage BC [11]. However, chemotherapy always causes severe side effects and toxicity in non-targeted tissues, such as fatigue, nausea, vomiting, hair loss, etc. Another side to the coin is drug resistance, the most challenging aspect of chemotherapy success. Therefore, many in vitro and in vivo studies have focused on increasing the effectiveness of chemotherapy while reducing its dose and side effects.

There is increasing evidence of an association between certain foods (such as alcohol, fruits, vegetables, meat, and soy foods) and the development and treatment of BC. Lifestyle factors such as diet, weight, and physical activity can significantly influence BC outcomes [12,13,14]. Establishing a dietary pattern based on high intakes of fruits, vegetables, whole grains, poultry, and fish and low intakes of sugars and high-fat dairy products can help women experiencing BC. It has been shown that adopting a balanced diet, particularly during chemotherapy, is important to ensure an adequate intake of energy and nutrients. In addition, a healthy lifestyle may also reduce the toxicity of anti-cancer therapies, improving their effectiveness and promoting women’s long-term health by reducing BC comorbidities (e.g., obesity, hypertension, hyperlipidemia, and diabetes) [15]. In this regard, over the past few years the co-use of chemotherapy and natural bioactive compounds has attracted great attention for the extraordinary antioxidant, anti-inflammatory, and antineoplastic properties of these compounds [16,17,18,19]. Phytochemicals are nutritional bioactive compounds found in different plant foods or dietary products and classified by their wide range of chemical and structural features, usually associated with diverse biological effects [20]. Some of these dietary compounds have been shown to have anti-angiogenic properties, specifically affecting molecular pathways and receptors involved in cell proliferation and restoring the tumor microenvironment [19]. Nevertheless, there are several challenges in phytochemical usage such as poor solubility, poor bioavailability, low stability, and sometimes the requirement of a high dosage. Phytochemicals differ in their solubility and heat resistance, impacting the quality of the products and their use in food and nutraceutical development [20]. For this reason, these compounds cannot be easily introduced through a balanced diet. Several studies, in fact, have been focused on different formulations to enhance the bioavailability and the therapeutic benefits of these dietary bioactive compounds. The most important advance in this research is the incorporation of these bioactive compounds into nanoformulations (characterized by low size with high surface/mass ratio and absorption), alone or together with conventional drugs, for more specific chemical delivery to cancer cells [21]. The origin of this nanotherapeutic approach was motivated by the enhanced permeability of the blood vessel endothelium in solid tumors and the ability of antitumor drugs and bioactive natural compounds with low molecular weights to cross this barrier in both directions, which prevents tumor tissues from efficiently accumulating these compounds. This feature of the cancer endothelium has inspired the research and creation of new drug delivery systems to target tumor cells effectively and passively [22,23,24]. In passive delivery systems, the drug gets encapsulated to protect it from being metabolized, which allows it to passively and slowly reach the target tumor tissue through the bloodstream. The active delivery systems instead exploit specific proteins, antibodies, and ligands on the surface of the delivery system. This system specifically binds to the surface antigen or receptor expressed by tumor tissue, triggering endocytosis in specific tumor cells. The most important and interesting aspect is that, while on the one hand, it is possible to enhance the antitumor effect of the drugs, on the other hand, it reduce the distribution of the drugs in normal tissues, limiting the possible onset of side effects. The last and most challenging drug delivery system is the physiochemical one based on the tumor microenvironment, mimicking biological responsiveness to control drug release at the target site.

In this review article, we will highlight some of the most significant dietary and phytochemical compounds that show significant anti-breast cancer activity both in vitro and in vivo.

A potential dietary strategy to reduce cancer risk is the use of polyunsaturated fatty acids (PUFAs), of which ω-3 and ω-6 are the two major classes. The terms ω-3 and ω-6 PUFAs refer to two groups of PUFAs that respectively contain a double carbon–carbon bond at the third carbon atom (*n*-3 position) and at the sixth (*n*-6 position) from the methyl end of the carbon chain. Historically, the intake of ω-3 and ω-6 PUFAs is estimated to be similar, but in recent years, the Western diet has led to a significant increase in ω-6 PUFA intake, leading to a growth in the ω-6/ω-3 PUFA ratio [25]. Excessive amounts of ω-6 or high ω-6/ω-3 ratios are linked to the development of various diseases, including cardiovascular, autoimmune, and some cancer diseases; on the other hand, increased amounts of ω-3 PUFA have been shown to have an inhibitory effect [25]. Previous studies have shown that ω-3 PUFAs competitively inhibit ω-6 fatty acids, thus lowering levels of inflammatory eicosanoids generated from ω-6 metabolism [14], and that higher ω-3 relative to ω-6 could reduce BC through inflammation, oxidative stress, and estrogen metabolism [26,27].

Another phytotherapeutic agent broadly used for its history of safe use is bromelain from pineapple extract. Bromelain has been used as a traditional folk medicine since ancient times as a treatment for severe wounds, edema, chronic rhinosinusitis, cardiovascular diseases, rheumatoid arthritis, and fibrinolytic affects, but only recently have its anticancer activity and immunomodulatory effects been highlighted [28]. Bromelain is a complex mixture of proteases, in particular thiol endopeptidases, able to interfere directly with cell surface receptors and several downstream pathways that support malignancy.

Isothiocyanate, also known as glucosinolate, is the most abundant naturally occurring dietary chemopreventive compound. Glucosinolates are abundant in cruciferous vegetables such as *Brassica oleracea*, watercress, Brussels sprouts, cabbage, Japanese radish, and cauliflower, and they significantly contribute to their cancer chemopreventive effect [29,30]. Upon consumption, glucosinolates are hydrolyzed by the plant enzyme myrosinase or in the colon through the actions of gut microorganisms in bioactive molecules such as indole-3 carbinol and sulforaphane [31]. Several studies have demonstrated that the ingestion of cruciferous vegetables may reduce overall tumor risk, especially for BC. In particular, several studies demonstrated that sulforaphane increases levels of detoxification enzymes and reduces cancer cell growth, inhibiting cell cycle, inducing apoptosis and autophagy, and removing cancer stem cells (CSCs) [32].

This review’s primary objective is to provide a comprehensive report on the most up-to-date scientific and clinical evidence available for these five primary phytochemical classes, many of which are currently being used as adjuvant therapeutics or chemopreventive agents in BC therapies at our hospital research institute.

## 2. Materials and Methods

An updated review of the role of PUFAs, bromelain, and glucosinolates in the chemoprevention of breast cancer was performed. Published studies were searched in specialized databases such as PubMed/MedLine, Scopus, Science Direct, and Google Scholar using the following keywords: “breast cancer”, “cancer prevention”, “phytochemicals”, “dietary bioactive compounds”, “polyunsaturated fatty acids”, “bromelain”, “glucosinolates”, “sulforaphane”, “indole-3 carbinol”, and “breast cancer cell lines” (Table 1). In particular, we focused on MCF-7 and T-47D as ER-positive cancer models, SK-BR-3 as HER2-positive cancer models, and MDA-MB-231 and MDA-MB-468 (PR-, ER-, and HER-2-negative cell lines) for triple-negative breast cancer (TNBC) studies. Lastly, MCF-10A cells are frequently used as a normal control in breast cancer studies.

Each article was published before May 2024 and written in English, and its significance was estimated by analyzing the title and abstract. Non-English text, lack of access to the full text, and publication in journals with no impact factor were considered exclusion criteria. Articles that met all search criteria were evaluated in the literature and included in this review.

## 3. Polyunsaturated Fatty Acids (PUFAs)

**Figure 1:**
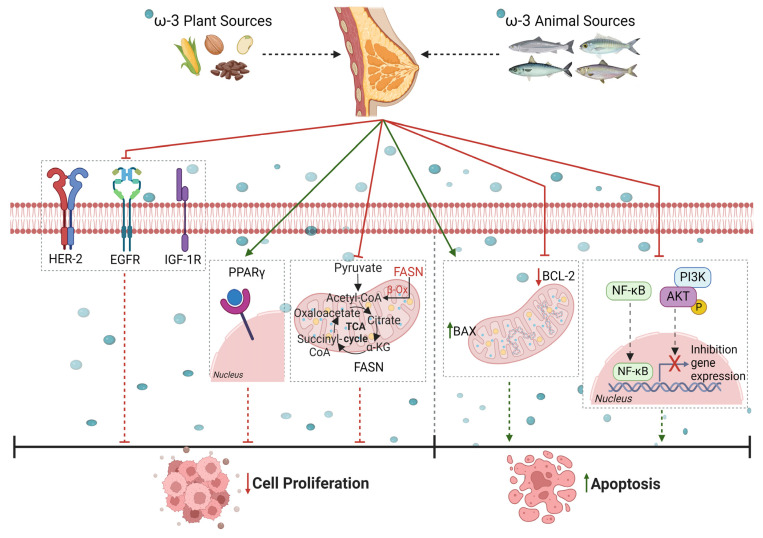
Scheme showing how ω-3 PUFAs inhibit (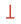) cell proliferation by reducing (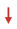) the expression of some growth factors, including human epidermal growth factor receptor-2 (HER2), epidermal growth factor receptor (EGFR), and insulin-like growth factor 1 (IGF-1R), by either activating (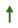) peroxisome proliferator-activated receptor gamma (PPARγ) or decreasing levels of fatty acid synthase (FAS) protein. Furthermore, PUFAs promote cell apoptosis by blocking phosphoinositide 3-kinase/protein kinase B (PI3K/Akt) pathways, downregulating phosphorylated Akt, inhibiting nuclear factor-kappa B (NF-κB) activity, and lowering B-cell lymphoma 2/B-cell lymphoma 2-like protein 4 (Bcl-2/Bax ratio). Created with BioRender.com (https://app.biorender.com/illustrations/65fc51a4a6668e33e3bf3d89 (accessed on 9 April 2024)).

Clinical and preclinical studies have shown that there is a relationship between the quantity and quality of dietary fat intake and tumor incidence and progress [33]. In mammals, both ω-6 and ω-3 PUFAs are essential for health and must be included in the diet, as they cannot be synthesized endogenously. Data obtained from experiments conducted in previous trials demonstrate that optimum amounts of ω-6 and ω-3 PUFAs can prevent or inhibit cell proliferation and/or induce apoptosis in various tumors. PUFAs, mainly ω-3 and ω-6, exert their inhibitory effects by acting as precursors of various bioactive lipids with anticancer activity [34], interfering with the regulation of cell growth, differentiation, apoptosis, and metastasis by affecting gene expression or signaling transduction pathways. Indeed, they exert antitumor effects in the following ways:

- Reducing the expression of some growth factors such as HER2, EGFR, and insulin-like growth factor 1 (IGF-1R);
- Inhibiting cell proliferation by activating peroxisome proliferator-activated receptor gamma (PPARγ) or decreasing levels of fatty acid synthase (FAS) protein;
- promoting apoptosis by blocking phosphoinositide PI3K/Akt pathways, Akt phosphorylation, and NF-κB activity and reducing the B-cell lymphoma 2/B-cell lymphoma 2-like protein 4 (Bcl-2/Bax) ratio (Figure 1).

### 3.1. ω-3 PUFAs

Alpha-linolenic acid (ALA, 18:3*n*-3) is the precursor of *ω*-3 PUFAs such as docosahexaenoic acid (DHA, 22:6*n*-3) and eicosapentaenoic acid (EPA, 20:5*n*-3). Because the human body has a limited ability to produce EPA and DHA from ALA, these PUFAs should be consumed through food sources. The major sources of ALA are plant oils, while those of EPA and DHA are marine animal products such as fish oils and other marine animal fats. They have important roles in human physiology and also exert beneficial effects on some chronic degenerative diseases such as cardiovascular diseases [35], rheumatoid arthritis [36], diabetes [37], several autoimmune diseases [38], and cancer [39]. ω-3 PUFAs have demonstrated an ability to chemosensitize BC tumors and thus potentially improve treatment efficacy. Therefore, the consumption of ω-3 PUFAs, for which fish is the greatest dietary source, may provide an opportunity to increase survival in women with BC [40]. Altogether, ALA, EPA, and DHA have several effects against breast cancer development in vivo. Another interesting aspect of *ω*-3 PUFAs is their use as ideal components of nanoformulations designed to be delivered more precisely to tumor tissues; because tumor cells grow at abnormal rates and require large amounts of FA to form their cell membranes, they necessitate and incorporate high quantities of ALA, EPA, and DHA [41]. Additionally, they have many double bonds, making them highly susceptible to peroxidation. Therefore, their inclusion in nanoformulations may help to protect them from oxidative degradation and improve their bioavailability and tumor specificity [21]. Recent studies investigated nanoformulations containing both doxorubicin (DOX) and ω-3 PUFAs to improve DOX delivery in BC. Recently, DOX was included in a mixed PUFA nanoformulation (MPUFAs-DOX@liposomes) containing a high amount of ω-3 PUFA, which showed higher toxicity than free DOX or DOX@liposomes in MCF-7 cells [42]. The authors attributed the enhanced anticancer activity to the increased lipophilicity of the nanoformulations, increased release rate of DOX from the ω-3 nanosystem, and enhanced uptake of DOX when delivered to cancer cells through the nanosystem [42].

#### 3.1.1. α-Linolenic Acid

ALA is a plant-derived ω-3 PUFA found in soybean oil (7.8%), canola oil (9.2%), and hemp oil (20%) [40], with important protective effects in BC. Dietary ALA supplementation inhibited the proliferation of breast cancer in xenograft rodent models with high levels of estrogen; indeed, ALA derived from flaxseed oil reduced MCF-7 cell proliferation and increased cell death [43] by downregulating tyrosine kinase receptors like EGFR and HER2, resulting in pAkt diminution. Further, in vitro studies also demonstrated that ALA reduced MCF-7 cell proliferation by 33% [43]. ALA from flaxseed oil showed a powerful ability to reduce the palpable tumor size when combined with tamoxifen, and it was also found to decrease HER2 expression and re-regulate growth factor signaling pathways by inhibiting IGF-1R and Bcl-2 [44]. A recent work showed that a nanoformulation made up of ALA and paclitaxel had a higher anti-BC effect in vitro and in vivo [45], since small hydrophobic molecules (like ALA) in nanoformulations allow for greater drug loading and for higher biocompatibility. So far, there are not many studies that support the inclusion of ALA in nanostructures because EPA and DHA show greater antitumor activity than ALA, but ALA advantages are often underestimated.

#### 3.1.2. Eicosapentaenoic Acid

EPA is a long-chain *ω*-3 PUFA that has 20 carbon atoms and five double bonds (20:5), whereas DHA has a longer chain, with 22 carbon atoms and six double bounds (22:6). DHA and EPA make significant contributions to the physical properties of biological membranes, such as membrane organization, ion permeability, elasticity, and eicosanoid composition. Among oily fish species, mackerel (1.8–5.3% by weight), herring (1.2–3.1%) and salmon (1.0–1.4%) contain a lot of EPA and DHA [16]. A higher intake of marine ω-3 PUFAs (EPA and DHA) was related to reduced BC relapse and all-cause mortality rates [46], but it is still unclear if EPA and DHA have different or similar effects on BC. In support of the specific effect of EPA on BC, various in vitro studies showed that EPA can induce BC cell apoptosis by inhibiting anti-apoptotic regulatory proteins, e.g., Bcl-2 [47], and by reducing total and phosphorylated Akt content in MCF-7 cells; moreover, in the same cells, combined treatment with EPA and tamoxifen enhanced the reduction in cancer proliferation. EPA-rich foods have also been shown to hinder breast cancer growth in animal and human studies. These inhibitory effects have been attributed to the high absorption of EPA by cancer phospholipids and subsequently to the disruption of inflammatory eicosanoid biosynthesis from arachidonic acid [48]. Recently, it has been proven that EPA regulates tumor growth in MCF-7 xenografts via the inhibitory G-mediated signaling pathway [49]. In addition, a clinical study in humans showed that plasma concentrations of EPA correspond to high levels of PPARγ mRNA in adipose tissue [50]. Because PPARγ activators can inhibit cell growth, the overexpression of PPARγ induced by EPA could be a potential mechanism of action, suggesting that EPA may act individually to hinder the growth and progression of BC.

#### 3.1.3. Docosahexaenoic Acid

Some studies have established the antitumor activity of DHA [51,52]. This activity involves influencing tumor proliferation, apoptosis, and differentiation and inhibiting angiogenesis [53], tumor cell invasion [54], and metastasis [55]. Barascu et al. [56] showed that EPA and DHA reduced MCF-7 and MDA-MB-231 cell growth and increased apoptosis, particularly for DHA, and specifically, they showed that ω-3 PUFA can hinder cell growth by blocking the cell cycle during the G2/M transition. HER2 signaling has an important role in many processes involved in cellular proliferation and survival [57]. In vitro studies have shown that DHA is able to disrupt lipid rafts in HER2-overexpressing cells, inhibiting the HER2 signaling pathway and therefore causing cell death [57]. Menéndez et al. [58] proved that DHA supplementation could downregulate the expression of the HER2/neu oncogene in SK-BR-3 and BT-474 cells, confirming the potential of DHA in HER2^+^ BC treatment. They also demonstrated that pre-exposure to DHA synergistically enhanced the cytotoxic effect of conventional drugs such as taxane and Taxol on highly metastatic BC cells [58] due to the fusion of DHA with cellular lipids, altering membrane fluidity and function and improving drug absorption [58]. Accordingly, human clinical trials have shown that DHA supplementation with chemotherapy improves survival in patients with metastatic BC [59]. They observed that time to progression (TTP) was significantly higher in patients with high DHA incorporation compared to patients with low DHA incorporation (8.7 months vs. 2.8 months). Moreover, they found that the overall survival (OS) was almost doubled in patients with high DHA incorporation (33 months vs. 17 months) [59]. It has also been hypothesized increased intracellular DHA levels induce the activation of apoptotic effector enzymes, e.g., caspase-8 and caspase-3, and the downregulation of Bcl-2, thereby causing apoptosis [60,61]. In vivo studies have also shown that DHA decreases the incidence of BC, coinciding with an increase in BRCA1 at the transcriptional and protein synthesis levels, which is an important sign of tumor suppression [62,63]. Nevertheless, recent studies have shown that DHA alone was not capable of reducing EGFR levels in MDA-MB-231 cells but enhanced EGFR inhibitors, indicating its potential in combined treatment [64]. DHA is also involved in the regulation of transcription factors such as NF-κB [65], activator protein 1 (AP-1) [66], c-myc [19], p53 [59,67], and PPARs. Taking a new perspective, it is thought that ω-3 PUFA can regulate microRNA expression in BC, e.g., miR-21. In this work, in fact, it was demonstrated that DHA reduces miR-21 levels, related to proliferation and metastasis development [68]. Metabolic dysfunction is one of the hallmarks of a tumor: cancer cells have altered energy production and rely heavily on aerobic glycolysis; thus, some of the main effectors of glycolysis can be used as tumor therapeutic targets [69]. Several studies have demonstrated that DHA can modulate various metabolic pathways in tumor cells by metabolic reprogramming, suggesting its potential implementation for the modulation of aerobic glycolysis and the Warburg effect [70]. Both parameters, the extracellular acidification rate (ECAR) and the oxygen consumption rate (OCR), which respectively indicate glycolysis and oxidative phosphorylation, significantly decreased in BT-474 and MDA-MB-231 cells due to DHA addition compared to those in untreated cells and non-tumorigenic control MCF-10A cells. These results indicate that, regardless of tumor cell types, DHA can alter the biological profile of tumor cells selectively by modifying mitochondrial structure and function, by activating AMPK protein, by decreasing ATP levels, by activating the LKB1 kinase protein, and by inhibiting hypoxia-inducible factor 1 (HIF1-α) [70,71]. One greatly researched nanoapproach for BC treatment regarding the combination of ω-3 PUFA and conventional drugs is the incorporation of DHA and DOX in nanostructures. DHA is often used to enhance the antitumor effects of DOX because it is deemed the most powerful ω-3 PUFA and is capable of enhancing induced cytotoxicity in cancer cells [72]. These nanostructures were used also in a BC spheroid model for the first time, showing an increase in cytotoxicity and a better penetration of doxorubicin [73]. Indeed, in a population-based follow-up study of women affected by BC, Khankari et al. [74] found that high intakes of DHA reduced all-cause mortality risk by 16% to 34% after 15 years of follow-up.

##### 3.2. ω-6 PUFAs

Linoleic acid (LA) and arachidonic acid (AA) are the two most common ω-6 PUFAs in typical Western diets. LA is found in some vegetable oils such as corn and safflower oils, while AA is usually derived from animal food sources or can be synthesized from LA [75]. ω-3 PUFAs are known to compete with LA, which is also known to be a main nutrient for tumors [41]. The ratio of these two PUFA classes are important because ω-3 and ω-6 have similar biological pathways and can compete with each other, creating an imbalance [76]. ALA represents a key molecule involved in the anti-inflammatory response. LA is associated with pro-inflammatory responses. Cancer development appears to be directly influenced by the dietary ω-3/ω-6 PUFA ratio. High levels of ω-6 PUFAs in Western countries have been associated with several types of tumor [41,77].

To quantitatively ascertain the relationship between the risk of BC and high intake of ω-3/ω-6 PUFAs, a meta-analysis of five cohort studies and six prospective nested case-control studies was performed [78]. According to reports, people with a higher intake of ω-3/ω-6 PUFAs had a lower risk of BC. Moreover, with an increment of about 1/10 in the ω-3/ω-6 PUFA ratio, a further 6% reduction in BC risk was observed. More importantly, a subgroup analysis has revealed that individuals with a higher adsorption of ω-3/ω-6 PUFAs in serum phospholipids had a 38% reduction in BC risk. Since EPA and DHA cannot be synthesized in mammals and conversion between ω-3 and ω-6 PUFAs does not occur in humans, serum phospholipid levels of ω-3/ω-6 PUFAs reflect their food absorption [79].

## 4. Bromelain

**Figure 2:**
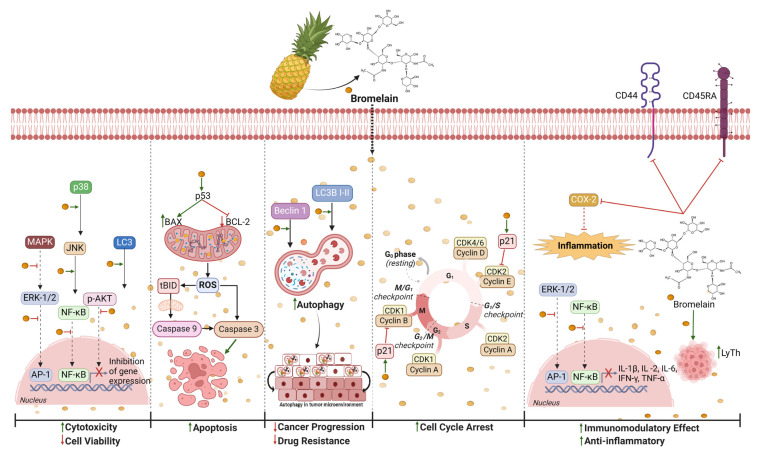
Suggested anticancer molecular mechanisms of bromelain. It induces (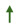) cytotoxicity and reduces (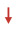) cancer cell proliferation primarily by (I) modulating the expression of genes crucial for cell differentiation and proliferation via the mitogen-activated protein kinase (MAPK) signaling pathway, extracellular signal-related kinase (ERK), nuclear factor-kappa B (NF-κB), c-Jun *N*-terminal kinase (JNK), serine/threonine protein kinase (Akt), microtubule-associated protein B-light chain 3 (LC3), and p38; (II) inducing cell death by apoptosis/autophagy via Bcl-2-associated X (BAX), B-cell lymphoma 2 (Bcl-2), caspases-3 and -9, reactive oxygen species (ROS), p53, LC3, and BECLIN 1; (III) blocking (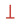) the cell cycle by inhibiting cyclins B and E through the activation of p21; (IV) having an immunomodulatory effect by reducing the production of IL-1β, IL-2, IL-6 (interleukin), interferon-gamma (IFN-γ), and tumor necrosis factor (TNF-α) by stimulating T helper cells (LyTh), and via the proteolytic cleavage of clusters of differentiation 44 (CD44) and CD45RA; and (V) reducing inflammation by influencing cycloox-ygenase-2 (COX-2). Created with BioRender.com. https://app.biorender.com/illustrations/6613c5688fba6222e90aa178 (accessed on 10 April 2024).

Pineapple (*Ananas comosus*) has been used since ancient times as medicine because of its anti-inflammatory and immunomodulatory effects (Figure 2). However, only recently has it been highlighted that the plant’s therapeutic qualities are primarily due to bromelain. Bromelain extract is a complex mixture of proteinase (commonly known as thiol proteinase or cysteine endopeptidase) and a smaller portion of non-protease components (such as phosphatases, glucosidases, peroxides, celluloses, glycoproteins, and carbohydrates) [80,81,82,83,84]. In biochemical terms, bromelain is a non-toxic compound with therapeutic values, classified as a protein-digesting enzyme protease [28]. Both the stem and the fruit of pineapple plants contain bromelain, yet the inedible stem contains a far higher amount of bromelain (around 80%) than the fruit (10%) [81,82,83,84]. Indeed, typically, stems are used to produce commercial bromelain using centrifugation, lyophilization, and ultrafiltration [80,84,85,86]. The stable secondary structure of stem bromelain has an optimal pH near neutrality and a maximal enzymatic activity at 30 °C [86]. The human body effectively absorbs its active form in the gastrointestinal tract, reaching a concentration of about 12 gm/day [83,87,88,89]. Bromelain is a good option for the development of future oral enzyme treatments in oncology because of all these qualities without any major side effects. Since its discovery, numerous in vitro and in vivo studies have shown that bromelain has various phytomedical activities: anti-inflammatory, fibrinolytic, anti-coagulative, antithrombotic, drug absorption-enhancing, and, most interestingly, anticancer actions [81,83,85,89]. Even though the anticancer effect is mainly due to enzymatic activity, some studies have highlighted that the non-enzymatic components of bromelain (polysaccharides, glycoproteins, vitamin C, β-carotene, and flavonoids) have a synergistic impact on the complex network involved [82,90]. Numerous studies have shown that, depending on the milieu, bromelain modulates the immune response in different ways. [28,83,91]. This is especially relevant in tumor development, where a network of secreted factors, infiltrating cells, stromal cells, fibroblasts, and inflammatory immune cells shape the tumor microenvironment (TME) [92,93]. The TME is a complex and continuously evolving entity where pro-tumorigenic inflammation and antitumor immunity promote cancer.

Various in vitro research models showed that bromelain caused a dose-dependent suppression of pro-inflammatory interleukin IL-6, IL-1β, and tumor necrosis factor alpha (TNF-α) production [94,95]. Bromelain treatment inhibited both NF-κB and MAPK pathways, resulting in a decrease in cyclooxygenases-2 (COX-2) and prostaglandin E2 (PGE2), two significant inflammatory mediators linked to the survival, invasion, proliferation, and immune escape of tumor cells [96,97,98]. A comparable anti-inflammatory impact of bromelain has additionally been noted, as decreasing extracellular signaling regulates kinase (ERK), c-Jun NH2-terminal kinase (JNK), p38 activation, and AP-1 transcription factor activity [99]. p38, JNK, and ERK pathways work together to phosphorylate and activate transcription factors, which in turn causes the expression of particular target genes in response to inflammatory stimuli [100,101]. However, environmental stress preferentially stimulates the p38 and JNK pathways, while mitogen stimuli primarily activate the ERK pathway. Extremely relevant is the effect of bromelain enzyme activity on immune superficial molecules/receptors. Immune cells treated with bromelain showed decreased expression of a vast array of superficial marker (cluster domain, CD) essential for adhesion, migration, and activation during inflammation [102,103,104]. In particular, the pro-inflammatory CD44 receptor expressed both in cancer cells and in immune cells is removed by bromelain action [94]. The downregulation of CD44 regulates lymphocyte homing and migration to the site of inflammation and is directly linked to tumor proliferation and progression. Furthermore, the presence of a proteinase inhibitor caused notable reduction in the proteolytic reactivity of bromelain against immune cell CDs [28]. Considerable evidence demonstrated the capability of bromelain to arrest cell proliferation or drive apoptosis or autophagy in GI-101A, MCF-7, and MDA-MB-231 triple-negative cells. Fouz et al. [105] have highlighted the antiproliferative effect of bromelain on MCF-7 cells. Interestingly, even when bromelain was encapsulated into lipid-core nanocapsules, the proteolytic and antiproliferative activities were preserved, producing a significantly greater impact than the bromelain solution [106]. Some years later, in two different studies by Raesi et al. [99,107] demonstrated dose-dependent antiproliferative and growth inhibition effects. MCF-7 cells seemed to be more resistant to lower concentrations of bromelain, with a growing sensitivity from an IC_50_ of 65 µg/mL [99] to a maximum of 100 µg/mL [108]. Significant results about how bromelain and cisplatin work together to synergistically improve the induction of death in MDA-MB-231 and MCF-7 cells were provided by Raesi et al. [107] and Pauzi et al. [109]. Referring to the study of Bhui et al. [110], it is important to highlight how bromelain drives both autophagy and apoptosis mechanisms. Bromelain induced autophagy and delayed apoptosis by phosphorylating JNK and p38 MAPKs, by reducing the phosphorylation of ERK1/2, and by suppressing autophagy marker levels (microtubule-associated protein 1A/1B-light chain 3 (LC3B II) and beclin-1) [110]. A similar effect was evaluated based on levels of pro-apoptotic proteins p53, p21, and Bax, which appeared upregulated, and anti-apoptotic proteins Bcl2, Bcl-x, and COX-2, which instead were downregulated [109,111,112]. The data provided by Dhandayuthapani et al. [113] are intriguing since they provides crucial details regarding the apoptosis promotion seen in GI-101A cells treated with bromelain. The findings of this investigation have shown that bromelain strongly increased the activity of caspase-3 and activated pro-caspase-9 to its active form [113]. Because ROS have multiple biological activities in numerous cellular processes and tumor development, it is challenging to attribute anticancer activity to ROS. Bhatnagar et al. carried out one of the earliest investigations on the pro-oxidative characteristics of bromelain on MCF-7 cells [111]. Bromelain increased ROS production, which caused oxidative stress and apoptosis. Additional studies revealed that bromelain increased ROS content, causing the mitochondrial membrane to depolarize, crucial in autophagy and cell apoptosis [114,115].

## 5. Glucosinolates

The scientific community’s interest in vegetables has been focused on preventing and reducing the risk of developing a tumor. Vegetables are a widely available and crucial components of the human diet, representing a significant proportion of nutritional intake in most countries, except for Western countries. Their high per capita consumption makes them a great source of vitamins, minerals, dietary fibers, and especially phytochemicals [116]. Numerous studies have shown that consuming cruciferous vegetables (broccoli, cabbage, cauliflower, and Brussels sprouts), which contain phytochemicals, might reduce the risk of developing cancer [117,118]. Cruciferous vegetables have many beneficial effects that can be attributed to the high content of sulforaphane (1-isothiocyanato-4-(methylsulfinyl)-butane) and indole glucosinolates. These compounds are metabolites derived from glucosinolates and play pivotal roles in various biological processes such as apoptosis, cell cycle arrest, autophagy, angiogenesis, and antioxidant and anti-inflammatory related signaling pathways and genes [117,118]. Glucosinolates derive from glucose and amino acids, and their side chains contain various modifications. In light of the chemical structure of the side chain, glucosinolates are divided into aliphatic (methionine, isoleucine, leucine, valine), aromatic (phenylalanine, tyrosine), and indole (tryptophan) groups. Glucosinolates are sulfur- and nitrogen-rich compounds, which upon hydrolysis by endogenous myrosinases enzymes that co-exist in the plant, generate a range of bioactive metabolites (e.g., isothiocyanates, thiocyanates, and nitriles) [119]. Glucosinolates, in particular glucoraphanin and glucobrassicin, are stored in vacuoles, protecting them from myrosinase degradation [119,120]. Indeed, glucoraphanin and myrosinase are located in different compartments of the plant. When the plant tissue is damaged, it triggers sulforaphane production [121]. Hence, in nature, the glucosinolate/isothiocyanate system serves a defensive mechanism against insects, pathogens, and herbivores [122]. Several factors, both endogenous and exogenous, can influence the concentration of glucosinolates. These include environmental temperature, radiation, soil fertility, water availability, tissue damage, and the type of tissue. Notably, higher glucosinolate concentrations are found in roots and seeds of younger tissue than in late vegetative tissue, whereas foliage displays moderate concentrations. Glucoraphanin can be found in all parts of broccoli plants, but it is primarily concentrated in the aerial sections, developing florets (flower buds), and seeds [123]. The level of glucoraphanin in broccoli seeds is largely influenced by genetic factors and the environment in which plants are grown, including location, year, drought, pollution, and disease pressure [124].

Cruciferae’s high content of glucosinolates is responsible for chemopreventive effects compared to other vegetables. However, when broccoli is cooked or blanched, the water-soluble glucosinolates are released into the water, affecting their content [122,125,126,127]. Hence, cooking methods that use less water, such as steaming or microwaving, may result in the retention of maximum sulforaphane content, in contrast to boiling [125,126,127]. For this reason, it is preferred to consume raw or freshly harvested broccoli. Even myrosinase is destroyed during meal preparation, being particularly sensitive to high temperatures and pH [128]. Unlike raw sprouts, many commercially available supplements lack myrosinase, which cannot be encapsulated because it requires freshness [129]. Bricker et al. [130] performed an in vivo study to observe how various cooking methods for broccoli sprouts or purified sulforaphane impact the conversion of glucoraphane to sulforaphane. The concentrations of sulforaphane metabolites in steamed broccoli sprouts were found to be significantly lower than those in non-heated sprouts. However, mild heating at 60 °C was observed to further increase sulforaphane metabolites in most tissues. The analysis of metabolite distribution in all tissues indicates the potential for systemic benefits of consuming cruciferous vegetable. Overall, this is an interesting finding that highlights the importance of proper cooking methods to maximize the nutritional value of vegetables. Atwell et al. [131] demonstrated that the consumption of myrosinase-treated broccoli sprout extract containing sulforaphane resulted in three times higher levels of sulforaphane metabolites in the plasma and urine of sprout consumers.

Isothiocyanate formation does not solely depend on plant myrosinase; enzymatic hydrolysis can also occur in the human upper digestive tract during the consumption of raw plants. Several studies have found that certain specific microbial strains, like cecal microbiota, possess myrosinase-like activity and glucoraphanin hydrolysis capabilities [132].

### 5.1. Sulforaphane

Sulforaphane is characterized by an electrophilic central carbon, which is part of the isothiocyanate group and can react with cysteine residues from other molecules and facilitate interactions with important signaling mediators [133] such as Nrf2, NF-κB, and inflammasome complexes.

**Figure 3:**
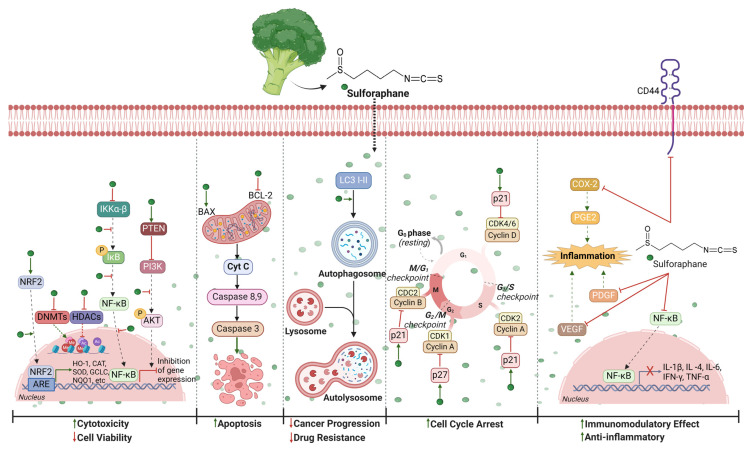
Suggested anticancer molecular mechanisms of sulforaphane. It induces (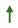) cytotoxicity and reduces (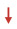) cancer cells proliferation primarily by (I) modulating the expression of genes crucial for cell differentiation and proliferation via the phosphatase and tensin homolog (PTEN), phosphoinositide 3-kinases (PI3K), and serine/threonine protein kinase (AKT) signaling pathways; via inhibitors of nuclear factor-kappa-B kinase subunit alpha and beta (IKK-α,β) and nuclear factor-kappa-B (NF-κB), inhibiting (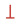) DNA methyltransferases (DNMTs) and histone deacetylases (HDACs); and via nuclear factor erythroid 2-related factor 2 (NRF2) by binding antioxidant response elements (AREs); (II) inducing cell death by apoptosis via Bcl-2-associated X (Bax); B-cell lymphoma 2 (Bcl-2); cytochrome complex (Cyt C); caspases 8, 9, and 3; and by autophagy via microtubule-associated protein light chain 3 I,II (LC3 I,II); (III) blocking the cell cycle by inhibiting cyclins D, A, and B through the activation of p21 and p27; (IV) having an immunomodulatory effect by reducing the production of IL-1β, IL-4, IL-6 (interleukin), interferon-gamma (IFN-γ), and tumor necrosis factor (TNF-α) and via the proteolytic cleavage of clusters of differentiation 44 (CD44); and (V) reducing inflammation by inhibiting cycloox-ygenase-2 (COX-2), prostaglandin E2 (PGE2), vascular endothelial growth factor (VEGF), and platelet-derived growth factor (PDGF). Created with BioRender.com. “https://app.biorender.com/illustrations/661e36e12bb8f001aab26646 (accessed on 16 April 2024)”.

Sulforaphane’s major target is Nrf2, a transcription factor that, after being activated, migrates into the nucleus and binds to antioxidant response elements (AREs), resulting in the enhanced expression of antioxidant and cytoprotective enzymes (e.g., NQO1, HO-1, glutamate-cysteine ligase catalytic subunit (GCLC), CAT, and SOD) (Figure 3). Licznerska et al. [134] found that Nrf2 expression increased after sulforaphane treatment, showing the highest increase in MCF-7 cells relative to MDA-MB-231 and MCF10-A control cells. Nrf2 antioxidant signaling, induced by sulforaphane, reportedly disrupts the NF-κB pathway [135]. Kim et al. [136] evaluated the synergistic effect of paclitaxel and sulforaphane treatment on MDA-MB-231 and MCF-7 cells. Sulforaphane’s presence in MDA-MB-231 cells prevented paclitaxel from activating NF-κB, expressing Bcl-2, and phosphorylating AKT. According to the authors, paclitaxel activates NF-κB through the classic signaling pathway, which involves IKK activation and IκBα degradation. The activation of IKK caused by paclitaxel was suppressed by sulforaphane treatment, which in turn suppressed the activation of NF-κB. In addition, sulforaphane and paclitaxel co-administration resulted in an improvement in paclitaxel-induced apoptosis through caspase-3, -8, and -9 while reducing Bcl-2 expression [136] (Figure 3). The regulation of inflammatory mediators, which are crucial for BC growth and progression, is a widely recognized function of NF-κB. Hunakova et al. [137] showed that sulforaphane inhibited the synthesis of pro-inflammatory cytokines (e.g., IL-1β, IL-6, TNF-α, IFN-γ), immunomodulatory cytokines (e.g., IL-4), and growth factors involved in angiogenesis (PDGF and VEGF) in a manner dependent on dosage.

To date, it has demonstrated that sulforaphanes in combination with classic chemotherapy drugs increase their effectiveness and safety. Xu et al. [138] showed that nanoparticles loaded with a mixture of cisplatin and sulforaphane decrease intracellular levels of glutathione via indirect sulforaphane Nrf2-activation. The significant reduction in glutathione was accompanied by a large rise in DNA-bound cisplatin and in death brought on by damage to DNA, restoring cisplatin chemosensitivity [138]. The effects of doxorubicin and sulforaphane were investigated by Rong et al. [139] using in vitro and in vivo studies. Usually, the tumor microenvironment is characterized by high levels of PGE2 (Figure 3). BC cells that are resistant to doxorubicin produce PGE2, which creates an immunosuppressive environment. Sulforaphane treatment can enhance the anticancer effects of chemotherapy drugs by blocking NF-κB and inhibiting the COX-2 level. Keshandehghan et al. [140] looked at the co-effects of sulforaphane and metformin on MCF-10A, MCF-7, and BT-474 cells. Although each compound has its own impact on cell progression and proliferation, when combined, their impact may be greater. The sulforaphane and metformin co-treatment had a greater impact on cell viability if there was an higher expression of HER2 mRNA; in particular, the co-treatment specifically impacted the Bcl-2/Bax ratio, cancer stem cell (CSC) genes, and CD44 expression. The development of drug carriers is aimed at improving the therapeutic success and preventing the systemic toxicity of chemotherapy. Pogorzelska et al. [141] developed a liposomal formulation combining doxorubicin and sulforaphane. Liposomes efficiently delivered both compounds into MDA-MB-231 and MCF-10A cells. Surprisingly, sulforaphane increased the amount of doxorubicin in the tumor cell nuclei. The co-treatment, in particular, caused a two-fold reduction in main cancer development, and doxorubicin concentration could be reduced by four times.

Besides its function in modulating the Nrf2/NF-κB signaling pathway, many studies have demonstrated that sulforaphane is a highly effective cell proliferation inhibitor, leading to apoptosis and cell cycle blocking. Kanematsu et al. [142] showed that different sulforaphane concentrations (1–100 µM) induced apoptosis in a manner dependent on dosage and timing in MCF-7 and MDA-MB-231 cells. In particular, sulforaphane caused S and G2/M cell cycle blocking linked to elevated p21 and p27 levels and lowered levels of cyclin A, cyclin B1, and CDC2 (Figure 3). In addition, an increase in caspase-3 and LC3-I and -II proteins, as well as the presence of autophagic vacuoles, triggered cell death. Lewinska et al. [143] in an interesting study demonstrated that sulforaphane (5–10 µM) promoted cell cycle arrest via p21 and p27 upregulation, while apoptosis was triggered at 20 µM. Sulforaphane caused accumulation at in the G2/M phase in MCF-7 and MDA-MB-231 cells, whereas it promoted arrest in the G0/G1 phase in SK-BR-3 cells. These data were subsequently confirmed by Cao et al. [144], who demonstrated that different concentrations of sulforaphane (0, 1, 2, 5, and 10 μM) hindered the viability of MCF-7 cells depending on dosage. In particular, sulforaphane decreased cyclin D1, involved in the cell cycle transition from the G1 to S phase, while it increased p21 and PTEN levels. Cancer cells rely on PTEN and PI3K/AKT pathways to regulate apoptosis and cell proliferation [145]. In particular, by phosphorylating p21, AKT stops the cell cycle at the G0/G1 phase [144]. Additionally, sulforaphane treatment reduced the expression of Bcl-2; upregulated Bax and cleaved caspase-3, -8, and -9; and significantly decreased CSC markers and CD44 [144] (Figure 3). Data consistent with those were obtained by Castro et al. [146], who tested sulforaphane’s effectiveness on mammosphere formation and the maintenance of breast cancer stem cells (BCSCs) isolated from MDA-MB-231-Luc-D3H1 cells. The ability of BCSC cells to form mammospheres decreased significantly after being administered sulforaphane, as indicated by their superficial markers CD44+/CD24−/CD49f+. This indicates a decreased capacity for these stem/progenitor cells to self-renew. Thus, phytochemicals are becoming new BCSC-eliminating agents, as well as future leads for drug development.

There is a widespread belief that sulforaphane is involved in epigenetic modulation, specifically by controlling the activation or silencing of specific genes and by attaching to DNA methyltransferases (DNMTs) and histone deacetylases (HDACs) [147,148] (Figure 3). Meeran et al. [149] demonstrated a considerable reduction in DNMT1 and DNMT3 levels by sulforaphane in MCF-7 and MDA-MB-231 cells but not in normal MCF-10A cells. Sulforaphane is a potent HDAC inhibitor, which suppresses the human telomerase reverse transcriptase (hTERT). This can lead to phenotypic changes in ER-negative cells, which become “ER enriched” [149]. Lewinska et al. [143] confirmed this epigenetic mechanism by detecting the upregulation of histone deacetylase 5 (HDAC5) mRNA levels in MCF-7, MDA-MB-231, and SK-BR-3 cells. However, the sulforaphane-treated MDA-MB-231 cells showed a reduction in HDAC3, HDAC4, HDAC6, HDAC7, HDAC8, HDAC9, and HDAC10 mRNA levels. The anticancer activity of sulforaphane is promoted by DNA hypomethylation, as shown by the reduced expression of DNMT1 and DNMT3B [143]. Li et al. [150] conducted a novel in vivo experiment that involved a prenatal/maternal mouse model to gain insight into how environmental factors, such as diet, can impact epigenetic programming and predict disease risk later in life. The data indicated that sulforaphane intake before birth shows a significant benefit in preventing the development of BC compared to treatment after birth. Prenatal and maternal sulforaphane intake had a significant impact on the levels and enzymatic activity of HDAC1, resulting in an active chromatin status and changing newborns gene expression profiles, possibly resulting in an increased risk of disease later in life. How early a person is introduced to dietary sulforaphane can have a major impact on how effective it is at preventing BC [150].

### 5.2. Indole-3 Carbinol

Among the glucosinolates, indole glucosinolates represent a major category. The breakdown of indole-3-methyl glucosinolate, also known as glucobrassicin, by myrosinase leads to the unstable intermediate form indole-3-methyl isothiocyanate, which is subsequently decomposed into indole-3-acetonitrile and indole-3-carbinol (I3C). I3C undergoes acid condensation in the gastric environment into dimers (3,3′-diindolymethane—sDIM), trimers, and tetramers [151,152]. A substantial body of evidence has been published on the healthful effects of I3C, including anti-inflammatory [153,154,155,156], neuroprotective [157,158,159], and anticancer activities [160,161,162,163,164]. Its proficiency in interfering with distinctive signaling pathways that regulate angiogenesis, apoptosis, cell cycle progression, estrogen signaling, and estrogen metabolism leads to its antitumor activity. In addition, the beneficial effects of I3C include its capacity to neutralize free radicals and inhibit lipid peroxidation, thereby reducing both hepatotoxicity and carcinogenesis [165,166,167]. These findings support the hypothesis that I3C may be also used as a chemopreventive and therapeutic agent in breast cancer.

According to a recent study, it was found that administering I3C at a concentration of 10 µM for 48 h effectively hampered cell growth in human breast cancer cells (MCF-7, MDA-MB-231) by triggering the apoptotic pathway (Caspase 3-4-8) [168]. The activation of the apoptotic pathway was attributed to the suppression of the Akt and NF-kB signaling pathways by I3C [169,170]. Moreover, there is strong evidence to suggest that I3C has antiproliferative properties that target specific components and regulators of the cell cycle. In a study conducted by Cover et al. [171], it was found that I3C can inhibit the growth of human MCF-7 cells via G1 cell cycle arrest by selectively inhibiting cyclin-dependent kinase 6 (CDK6) activity at both the transcriptomic and protein levels. Additionally, this reduction is associated with a decrease in endogenous retinoblastoma (Rb) phosphorylation. The expression of CDK2 and CDK4 was not affected by I3C treatment. Analogously, I3C signaling also suppressed breast cancer cell growth and reduced CDK6 production in estrogen-dependent and estrogen-independent ways [171]. The selective downregulation of CDK6 expression following indole I3C treatment was further confirmed at the mechanistic level: it was demonstrated that I3C interacts with the Sp1 transcription factor binding sites within the CDK6 gene promoter region, resulting in a loss of cellular CDK6 activity [172]. Furthermore, the upregulation of the p21Waf1/Cip1 CDK inhibitor was also reported to be a significant effect of I3C [173]. The findings of cell growth suppression were further confirmed in MCF7 cells, where I3C treatment (100 µM) was efficient in suppressing cell growth and causing a cell cycle shift to G1. The antiproliferative effect was further enhanced when I3C was used in combination with tamoxifen (1 µM), a well-known estrogen receptor antagonist, providing evidence of its potential use for the management of estrogen-responsive breast cancer subtypes. The protein levels of CDK6 and the p21 CDK inhibitor were found to be decreased and increased, respectively, following IC3 treatment. In contrast, tamoxifen did not affect the levels of these cell cycle proteins [174]. The effectiveness of I3C was also investigated in vivo in mouse models. A study conducted by Hajra et al. [175] assessed the antitumor activity of I3C monotherapy (20 mg/kg b.w.) and its ability to enhance the effect of doxorubicin (5 mg/kg b.w. i.p.), an anthracycline drug, in an animal model of breast adenocarcinoma (Erlich ascites carcinoma). The treatment was administered by feeding mice on two schedules: a concomitant treatment schedule (10 days of treatment after cell inoculation) and a pretreatment schedule (10 days of treatment after cell inoculation preceded by pretreatment for 15 days). The study revealed that the co-administration of I3C and doxorubicin on both treatment schedules resulted in the highest tumor growth inhibition (59.74% and 69.48%, respectively, in comparison to the tumor control group); moreover, increased levels of oxidative stress markers (ROS, NOS, lipid peroxidation), apoptotic index, and DNA damage in tumor cells were also reported. Additionally, I3C exhibited a cardioprotective effect by reducing doxorubicin-induced cardiotoxicity (ROS, NOS, LPO) and decreasing the expression of pro-inflammatory mediators (COX-2, iNOS, IL-6). The cardioprotective effect was also demonstrated in creatine phosphokinase and creatine kinase MB decreases. Furthermore, I3C also promoted the activity of antioxidant enzymes and inhibited peritoneal neovascularization by downregulating VEGF-A and MMP-9 [175]. A study conducted by Wang X. et al. [176] also evaluated the effect of a pairwise combination screening of two phytochemicals, I3C and luteolin (3′,4′,5,7-tetrahydroxyflavone), on breast cancer proliferation [176]. The biological assessment of I3C and luteolin, both individually and in combination, was conducted on ERα+ cells (MCF-7 and T-47D) and TNBC cells (MDA-MB-231 and BT-549). Individual agents inhibited BC cell proliferation in a dose-dependent manner. However, the inhibitory concentration was a higher molar concentration, whereas their combination (L30I40) demonstrated a synergistic antiproliferative effect on ER+ cells at a lower molar concentration of 30 µM and 40 µM, respectively. The biological effect was achieved via the downregulation of CDK4/6 and the induction of apoptosis. Furthermore, the combination treatment was further assessed in an animal model of MCF-7 cell-derived xenograft mice. A significantly lower tumor weight and tumor size were reported in the combination group with LUT and I3C (LUT10 mg + I3C10 mg/kg/day) compared to those in the control treatment (vehicle, alone treatments), suggesting L30I40 treatment as a chemotherapeutic approach for the treatment of ER^+^ BC subtypes [176]. The therapeutic potential of I3C against breast cancer was revealed by Rahman et al. [177] in a study conducted on non-tumorigenic and tumorigenic breast epithelial cells, MCF-10A and MCF-10CA1a, respectively, which revealed that I3C selectively induced apoptosis in the breast cancer cells by upregulating the Bax/Bcl-2 ratio and downregulating Bcl-XL expression [177]. According to Caruso et al. [178] a wide range of ER-α^+^ cells (MCF-7, ZR-75-1, T-47D) were more sensitive to I3C’s antitumor effects than ER-α-negative breast cancer cells (MDA-MB-231, MDA-MB-157, MDA-MB-436) and immortalized human mammary epithelial cells (HMECs). The study also found that I3C impacts several molecular mechanisms, including hormone receptor signaling (ERα, PR, GATA3, AR), increased apoptosis (cleaved caspase 3, SMAC, IAP), p53-signaling, and oxidative stress (ATF-3), as well as the arrest of cell proliferation (p21, c-myc, Rb pS807/S81) in ERα-sensitive cells compared to ER-α-negative cells [178]. In addition to the above, among the cellular signaling pathways that were also modulated by I3C supplementation was that of aryl hydrocarbon receptor (AhR) [179,180,181]. AhR belongs to the nuclear receptor superfamily and is a ligand-activated receptor that, upon translocation into the nucleus, activates genes linked to inflammation, detoxification, and cancer [179,182]. The anticancer activity of IC3, including mammosphere formation inhibition, has been evaluated in cancer stem cells derived from breast cancer MCF-7 cells in vitro [183]. The molecular mechanistic model of AhR activation was evaluated for several structurally diverse AhR agonists, including 3-methylcholanthrene (3MC), benzo[a]pyrene (BaP), 7,12-dimethylbenz[a]anthracene (DMBA), kynurenine (KYN), 6-formylindolo [3,2-b]carbazole (FICZ), indole-3-acetic acid (IAA), and indole derivatives such as indole-3-carbinol (I3C) and indirubin, in MCF-7 cells genetically engineered for the AhR gene (AhR-wild type/knockout in MCF-7 cells) [183]. Each AhR agonist demonstrated a characteristic suppression of mammosphere formation, which was observed to be dose- and AhR-dependent. Moreover, at a higher concentration (100 µM) of I3C and IAA, similar suppression levels of mammosphere formation were observed in AhR-WT and in AhR-KO cells, without impaired cytotoxicity [183]. Additionally, the activation of xenobiotic transcription factors was also evaluated at the transcriptional level. The upregulation of genes associated with xenobiotic metabolism was assessed in [183,184] after I3C treatment and suggests that I3C can modify drug efficacy and safety. Moreover, further evidence has been provided to support the hypothesis that the AhR signaling pathway plays a role in promoting the anticancer effect of I3C in ER-α^+^ cell lines [178]. Several AhR target genes (CYP1A1, CYP1A2, ALDH1A3, ALDH3A1) were upregulated in ER-α^+^ cells (MCF-7) after I3C administration, compared to expression in ER-α-negative cells at the transcriptomic level (microarray). Furthermore, the sensitivity to I3C was found to be reduced following the AhR knockdown of MCF-7 cells [178]. Numerous studies have revealed that I3C has the capability to reduce the growth of BC cells by regulating microRNA expression (miRNA) [185,186] and by modulating the epigenetic mechanism [187]. Hargraves et al. [185] discovered that I3C’s antitumor effects are closely associated with raised levels of miR-34a expression, which rely on the existence of the p53 protein. In particular, in human breast cancer cells (MCF-7) possessing wild-type p53, I3C triggered a dose-dependent surge in miR-34a expression, as well as the activation of the phosphorylated form of p53 at serine-15. However, the induction of miR-34a expression by I3C was blocked when mammary epithelial cells (MCF-10A) were transfected with a dominant negative p53. Furthermore, I3C was found to restrain the proliferation of MCF-7 cells by arresting the cell cycle at the G1 phase when used at a concentration of 200 µM [185]. According to research conducted by El-Daly et al. [186], the use of I3C in MCF-7 cells can influence the epigenetic network of tumor suppressor microRNAs and oncogenic microRNAs. This, in turn, impacts critical signaling pathways that control cell cycle progression (CDK4, CDK6) and the anti-apoptotic pathway (Bcl-2, survivin) via their suppression. The study discovered that after 72 h of I3C treatment, tumor suppressor microRNAs’ expression levels increased (let-7a-e, miR-17-5p, miR-19a, miR-20a). Conversely, there was a decrease in the expression of oncogenic microRNAs (miR-181a/b, miR-210, miR-221, and miR-106a) observed both at 48 h and 72 h [186]. Similarly, the interaction between epigenetic modulation, predominantly acetylation-based, and the administration of the dietary bioactive compound has been also evaluated in TNBC (MDA-MB-231, BT-549, HCC70, HCC1806), which lacks effective treatment options. The administration of an epigenetic modifier, vorinostat, and the dietary bioactive compound I3C, both used as single agents, was found to induce a re-expression of the ER-α and PR receptors. Furthermore, I3C was demonstrated to enhance the anticancer effect of vorinostat, inhibiting cell growth and invasion through an estrogen-independent mechanism in HCC70 and HCC1806 breast cancer cells [187]. Due to its instability in an acid milieu, such as the gastric juice, and its consequent rapid oligomerization in 3,3′-diindolylmethane (DIM), encapsulation technology has been proposed as a promising approach to increase its stability and prevent its degradation [188,189]. Among such approaches, Gehrcke et al. [190] proved the cytotoxic effect of free I3C and I3C embedded in nanocapsules of poly-(ε-caprolactone), prepared using rosehip oil (RHO), on the breast adenocarcinoma MCF-7 cell line. A higher cytotoxic effect was reported for I3C embedded in nanocapsules (85%) than for free I3C (50%) by using a sulforhodamine B assay. Furthermore, the encapsulation technology also increased I3C photostability [190]. The aforementioned findings elucidate the therapeutic potentiality of I3C, either alone or in combination with chemotherapeutic or epigenetic drugs, as supported by experimental in vitro and in vivo evidence.

Among the condensation products of I3C, DIM is the primary bioactive in vivo compound [188]. The analysis of plasma samples from a female population at elevated risk of breast cancer following the oral administration of I3C revealed the presence of DIM at all tested doses of I3C, ranging from 400 mg to 1200 mg. Conversely, due to its high instability, I3C was undetectable in plasma samples regardless of the I3C dose. Therefore, DIM was the sole condensation product detected in human plasma [191]. The antitumor effectiveness of DIM was also evaluated in breast cancer cell lines. In human MCF-7 and T47-D cell lines, DIM was found to induce apoptosis [192,193] and inhibit breast cancer cell growth. Specifically, DIM was effective in MCF-7 and MDA-MB-231 cell lines in a concentration- and time-dependent manner [193,194]. Several lines of evidence support its ability to impair breast cancer proliferation through the inhibition of multiple signaling pathways [195,196,197,198,199,200]. Furthermore, the modulatory effect of DIM on miRNA expression was investigated in human breast cancer cells [186,201,202] and in breast cancer organoids [203]. Moreover, to enhance the bioavailability of DIM, nanoformulations of DIM were examined. These included DIM nanoparticles coated with PEG/chitosan [204] and DIM-chitosan [205]. The results demonstrated enhanced in vitro antitumor activity against breast cancer cells, as evidenced by a significant reduction in cell migration and angiogenesis and a marked increase in apoptosis [204].

## 6. Conclusions and Future Prospects

Cancer remains a global challenge. Common treatment for BC includes hormonal therapy, surgery, targeted therapy, radiation therapy, and chemotherapy. Researchers have been motivated to find an alternative treatment, as standard therapies are correlated with the onset of side effects and poor surgical healing. Moreover, cancer cells gradually have started to exhibit drug resistance, hindering the therapeutic effect. To overcome these multiple obstacles of conventional treatments, there is a pressing need for new therapeutic, adjuvant, or chemopreventive compounds.

In recent decades, the use of medicinal plants, herbs, and dietary products with pharmacological significance has been rediscovered. What is especially intriguing are plant-derived small molecules (phytochemicals) used in traditional medicine for decades, which have been demonstrated to have remarkable antitumor activity. The protective action of certain dietary micronutrients, namely PUFAs, bromelain, sulforaphane, and indole-3-carbinol, against BC by inhibiting proliferation, invasion, angiogenesis, and metastasis has been well documented.

BC is now largely understood to be a heterogenous disease that is characterized by mutations in different sets of genes and pathways. Dietary bioactive compounds have shown multi-targeted “pleiotropic” effects with the ability to interfere with multiple oncogenic signaling pathways involved in the regulation of different stages of breast cancer development, such as PI3K/Akt/mTOR, JAK/STAT, MAPK, and NF-κB pathways; cell cycle and apoptosis; proliferation; metastasis; and angiogenesis. A thorough understanding of its multiple antitumor effects could lay the foundation for future research and comprehension to improve its clinical utility as well as applications in primary (preventing cancer), secondary (preventing potential metastasis growth), and tertiary (for cascading problems) care.

Unfortunately, the poor solubility and low bioavailability of these compounds limit their direct use in clinical applications. However, technological advancements and alternative therapeutic approaches have overcome these challenges. A variety of nanoformulations are now being used in a great range of biomedical applications, including large-scale production, stability, bioavailability, and quality control of drug loading and release.

Bromelain, ω-3 PUFAs, sulforaphane, and indole-3-carbinol are considered promising approaches to treating BC because of their reduced toxicity and side effects, making them ideal co-adjuvant supplements for traditional drugs and breast cancer chemoprevention tools. In conclusion, the use of dietary compounds and phytochemicals as part of standard treatment for BC requires further investigation.

## Figures and Tables

**Table 1:** Summary of non−cancerous and malignant breast cancer cells lines and their molecular classification.

Cell Lines	Organism	Immunoprofile	Characteristics
BT−474	Human	ER^−^, PR^+^, HER2^+^	Epithelial cell line from ductal carcinoma
BT−549	Human	ER^−^, PR^−^, HER2^−^	Epithelial cell line from ductal carcinoma
GI−101A	Human	ER^−^, PR^−^, HER2^+^ enriched	Epithelial cell line from a metastatic breast tumor
HCC70	Human	ER^−^, PR^−^, HER2^−^	Epithelial cell line from ductal carcinoma
HCC1806	Human	ER^−^, PR^−^, HER2^−^	Epithelial cell line from mammary gland
MCF−10A	Human	ER^−^, PR^−^, HER2^−^ and EGFR^+^	Non-tumorigenic epithelial cell line from mammary gland
MCF−10F	Human	ER^−^, PR^−^, HER2^−^ and EGFR^+^	Non-tumorigenic epithelial cell line from mammary gland
MCF−7	Human	ER^+^, PR^+^, HER2^−^	Epithelial cell line from mammary adenocarcinoma
MDA−MB−231	Human	ER^−^, PR^−^, HER2^−^ and EGFR^+^	Epithelial cell line from mammary adenocarcinoma
MDA−MB−453	Human	ER^−^, PR^−^, HER2^+^ enriched and AR^+^	Epithelial cell line from metastatic mammary carcinoma
MDA−MB−468	Human	ER^−^, PR^−^, HER2^−^ and EGFR^+^	Epithelial cell line from mammary adenocarcinoma
SK−BR−3	Human	ER^−^, PR^−^, HER2^+^ enriched	Epithelial cell line from mammary adenocarcinoma
SUM−159PT	Human	ER^−^, PR^−^, HER2^−^	Epithelial cell line from mammary carcinoma
T−47D	Human	ER^+^, PR^+^, HER2^−^	Epithelial cell line from infiltrating ductal carcinoma
ZR−75−1	Human	ER^+^, PR^+^, HER2^−^	Epithelial cell line from ductal carcinoma

ER (estrogen receptor), PR (progesteron receptor), HER2 (human epidermal growth factor receptor 2), EGFR (epi-dermal growth factor receptor), and AR (androgen receptor).
